# Bradykinesia in idiopathic normal pressure hydrocephalus evaluated by quantitative finger tapping test: preliminary study

**DOI:** 10.1186/2045-8118-12-S1-P38

**Published:** 2015-09-18

**Authors:** Namiko Nishida, Yuko Sano, Akihiko Kandori, Hiroki Toda, Sadayuki Matsumoto, Koichi Iwasaki, Masatsune Ishikawa

**Affiliations:** 1 Department of Neurosurgery, Tazuke Kofukai Foundation, Medical Research Institute and Kitano Hospital, Japan; 2 Hitachi Ltd. Research & Development Group, Center for Technology Innovation - Healthcare, Japan; 3 Department of Neurology, Tazuke Kofukai Foundation, Medical Research Institute and Kitano Hospital, Japan; 4 Department of neurosurgery and normal pressure hydrocephalus center, Rakuwakai Otowa Hospital, Japan

## Introduction

Movement disorders in idiopathic normal pressure hydrocephalus (iNPH) are represented by gait disturbance (i.e., lower body bradykinesia). However, upper extremity bradykinesia was frequently found among iNPH patients. We assessed their upper extremity function by quantitative finger tapping test and checked the correlation with other demographic factors.

## Methods

We evaluated the 10-second finger tapping movements of 8 patients (age: 78.4 ± 3.8 y; males: 5, females: 3) using magnetic-sensor coil system. Clinical symptoms were evaluated by the iNPH grading scale, mini-mental state examination and frontal assessment battery (FAB). The correlation of tapping parameters with clinical indicators was estimated.

## Results

The patient's age correlated significantly with 6 of 21 finger-tapping parameters, including total tapping distance (Spearman r = -0.82, p = 0.013), coefficient of variation of maximum amplitude (r = -0.78, p = 0.023), energy balance (r = -0.72, p = 0.046), average maximum opening acceleration (r = -0.75, p = 0.034), tapping frequency (r = -0.85, p = 0.005), and average finger tapping interval (r = 0.87, p = 0.007). The severity of illness represented by iNPH grading scale correlated with other 2 parameters, including average maximum closing velocity (r = -0.73, p = 0.043) and coefficient of variation of maximum closing velocity (r = -0.79, p = 0.047).

## Conclusions

Our data support the diagnostic value of quantitative finger tapping test for estimating the severity of bradykinesia underlying the iNPH symptomatology. Though preliminary, different patterns of correlations found in this study could potentially indicate the fundamental processes discriminating aging and disease progression.

